# Adaptation and Diagnostic Potential of a Commercial Cat Interferon Gamma Release Assay for the Detection of *Mycobacterium bovis* Infection in African Lions (*Panthera leo*)

**DOI:** 10.3390/pathogens11070765

**Published:** 2022-07-04

**Authors:** Rachiel Gumbo, Tashnica T. Sylvester, Wynand J. Goosen, Peter E. Buss, Lin-Mari de Klerk-Lorist, O. Louis van Schalkwyk, Alicia McCall, Robin M. Warren, Paul D. van Helden, Michele A. Miller, Tanya J. Kerr

**Affiliations:** 1 DSI-NRF Centre of Excellence for Biomedical Tuberculosis Research; SAMRC Centre for Tuberculosis Research; Division of Molecular Biology and Human Genetics, Faculty of Medicine and Health Sciences, Stellenbosch University, Tygerberg 7505, South Africa; rachy@sun.ac.za (R.G.); tashnicao@sun.ac.za (T.T.S.); wjgoosen@sun.ac.za (W.J.G.); rw1@sun.ac.za (R.M.W.); pvh@sun.ac.za (P.D.v.H.); tjkerr@sun.ac.za (T.J.K.); 2 South African National Parks, Veterinary Wildlife Services, Kruger National Park, Skukuza 1350, South Africa; peter.buss@sanparks.org; 3 Skukuza State Veterinary Office, Department of Agriculture, Land Reform and Rural Development, Skukuza 1350, South Africa; linmariedk@dalrrd.gov.za (L.-M.d.K.-L.); lvs@vodamail.co.za (O.L.v.S.); 4 Department of Veterinary Tropical Diseases, Faculty of Veterinary Science, University of Pretoria, Onderstepoort, Pretoria 0110, South Africa; 5 Department of Migration, Max Planck Institute of Animal Behavior, 78315 Radolfzell, Germany; 6 Hluhluwe State Veterinary Office, Kwazulu-Natal Department of Agriculture and Rural Development, Hluhluwe 3960, South Africa; bugmccall@gmail.com

**Keywords:** African lion, bovine tuberculosis, cytokine ELISA, IFN-γ, interferon-gamma release assay, *Mycobacterium bovis*, *Panthera leo*

## Abstract

*Mycobacterium bovis* (*M. bovis*) infection in wildlife, including lions (*Panthera leo*), has implications for individual and population health. Tools for the detection of infected lions are needed for diagnosis and disease surveillance. This study aimed to evaluate the Mabtech Cat interferon gamma (IFN-γ) ELISA^Basic^ kit for detection of native lion IFN-γ in whole blood samples stimulated using the QuantiFERON^®^ TB Gold Plus (QFT) platform as a potential diagnostic assay. The ELISA was able to detect lion IFN-γ in mitogen-stimulated samples, with good parallelism, linearity, and a working range of 15.6–500 pg/mL. Minimal matrix interference was observed in the recovery of domestic cat rIFN-γ in lion plasma. Both intra- and inter-assay reproducibility had a coefficient of variation less than 10%, while the limit of detection and quantification were 7.8 pg/mL and 31.2 pg/mL, respectively. The diagnostic performance of the QFT Mabtech Cat interferon gamma release assay (IGRA) was determined using mycobacterial antigen-stimulated samples from *M. bovis* culture-confirmed infected (*n* = 8) and uninfected (*n* = 4) lions. A lion-specific cut-off value (33 pg/mL) was calculated, and the sensitivity and specificity were determined to be 87.5% and 100%, respectively. Although additional samples should be tested, the QFT Mabtech Cat IGRA could identify *M. bovis*-infected African lions.

## 1. Introduction

Large African carnivores, including lions (*Panthera leo*), are considered keystone species within conservation areas, because of their crucial role as apex predators in the ecosystem and their economic contribution to the tourism industry [1,2]. However, lions are listed as vulnerable by the International Union for Conservation of Nature (IUCN), since most of their subpopulations are decreasing [3]. Infectious diseases such as those caused by *Mycobacterium bovis*, feline immunodeficiency virus, and canine distemper virus may pose additional threats to lions, and potentially impact already shrinking lion populations [4,5,6].

*Mycobacterium bovis* is a member of the *Mycobacterium tuberculosis* complex (MTBC) and is the causative agent of bovine tuberculosis (bTB) [7]. Tuberculosis has a broad impact on human, domestic, and wild animal health due to the zoonotic and anthroponotic nature of MTBC, and threatens public health, food security, and livelihoods of farmers and ranchers who rely on animal production, game sales, and tourism as their primary source of income [8]. In South Africa, African buffaloes (*Syncerus caffer*) are one of the wildlife maintenance hosts of bTB, with spill-over to lions through predation on infected prey species [9]. In addition, *M. bovis* transmission may occur within a pride due to fighting, grooming, other social behaviour, and shared feeding at carcasses [10]. Importantly, introduction of *M. bovis* into naïve populations may have health and conservation consequences [9].

The development of tools for early detection of *M. bovis* infection in lions is essential for disease surveillance, individual diagnosis, and the screening of animals for translocation [8]. However, obtaining sufficient reference samples for the validation of diagnostic tests for wildlife species is challenging [11]. Currently, the only widely available ante-mortem test for the diagnosis of *M. bovis* infection in lions is the tuberculin skin test (TST). However, this test is considered impractical in free-ranging animals since it requires locating lions and performing two immobilisations 72 h apart [12,13]. As an alternative, a novel blood-based mycobacterial antigen-specific chemokine (C-X-C motif ligand 9; *CXCL9*) gene expression assay (GEA) was developed for identifying *M. bovis*-infected African lions [14]. Although this research assay only requires a single capture, it is costly and requires specific technical expertise and specialised equipment which may not be readily available. Serological tests to detect antibodies to *M. bovis* have been evaluated in lions, although results suggested insufficient sensitivity in lions with subclinical *M. bovis* infections [13]. Therefore, ancillary blood-based immunological assays need to be explored.

A previous report has described the development of a lion interferon gamma release assay (IGRA), which used monoclonal antibodies developed specifically to lion interferon gamma (IFN-γ). However, the assay was not evaluated using samples from known *M. bovis*-infected lions [15]. More recent studies have shown that commercial anti-domestic cat (*Felis catus*) IFN-γ antibodies could recognize native cheetah (*Acinonyx jubatus*) IFN-γ and lion recombinant IFN-γ (rIFN-γ) [16,17]. The implementation of a cross-reactive commercially available IGRA for bTB detection in lions would facilitate rapid diagnosis and provide a test for screening translocation candidates. Therefore, the aims of this study were to (a) assess cross-reactivity between commercially available antibodies to domestic cat IFN-γ and native African lion IFN-γ using mitogen-stimulated samples, and (b) evaluate the test performance of the QuantiFERON^®^-TB Gold Plus (QFT) Mabtech Cat IGRA kit for detecting antigen-specific immune responses in African lions.

## 2. Results

Initial screening with the Mabtech Cat IFN-γ ELISA^Basic^ kit detected high relative IFN-γ concentrations in African lion plasma from mitogen-stimulated (QFT mitogen plus Pokeweed Mitogen (PWM)) blood samples (Table 1), with minimal background signal observed in plasma from unstimulated (QFT nil) lion blood samples. In addition, a significant difference was observed between IFN-γ concentrations in QFT mitogen and QFT nil lion plasma samples (*p* = 0.0001), at all tested dilutions (Table 1). Based on the high calculated differential IFN-γ concentration, a 1:4 plasma dilution was chosen for all subsequent assays to preliminarily validate and determine diagnostic performance of the Mabtech Cat IFN-γ ELISA^Basic^ kit used with African lion QFT plasma samples.

### 2.1. Testing the Diagnostic Potential of the QFT Mabtech Cat IGRA for African Lions

**Figure 1:**
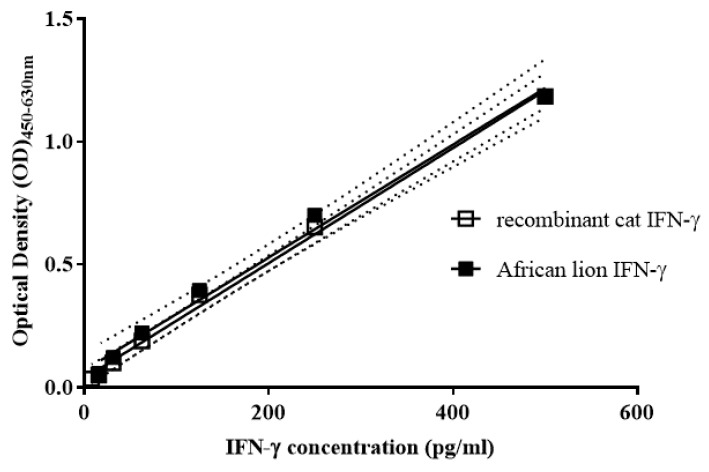
Linear regression analysis of a dilution series of domestic cat recombinant interferon gamma (rIFN-γ) and African lion IFN-γ (*n* = 3) with concentrations between 15.6 and 500 pg/mL, measured using the Mabtech Cat IFN-γ ELISA^Basic^ kit. Standard error is indicated by the dotted lines. Both sample types displayed linearity (R^2^ > 0.99), and no significant difference was observed between slopes for the domestic cat rIFN-γ and lion IFN-γ samples (F = 0.12; *p* = 0.73).

Linearity between optical density (OD) values and IFN-γ concentrations (pg/mL) was observed for both domestic cat recombinant IFN-γ (rIFN-γ) and three endogenous African lion IFN-γ samples with concentrations between 15.6 and 500 pg/mL (R^2^ > 0.99; Figure 1). In addition, the difference between the slopes of the dilution series of domestic cat rIFN-γ and African lion IFN-γ was not statistically significant (F = 0.12; *p* = 0.73; Figure 1).

The calculated IFN-γ concentrations for 15 unstimulated (QFT nil) African lion plasma samples used to create a matrix pool were 0 pg/mL (data not shown). The mean recovery values of spiked domestic cat rIFN-γ in lion plasma matrices are shown in Table 2. Recovery improved as the plasma matrix was diluted, with recovery in the 50% and 25% lion plasma matrices within the acceptable range (80–120%). Therefore, a 1:4 plasma dilution was chosen for all subsequent assays.

The coefficients of variation (CV%) for intra-assay (within-run repeatability) and inter-assay precision (between-run reproducibility), using QFT mitogen samples from three African lions, were 1.9–5.1% and 5.0–9.3%, respectively (Table 3). The observed CVs fell within the acceptable ranges of <10% for repeatability and <15% for reproducibility. The calculated limit of detection (LOD) and limit of quantification (LOQ) values for the assay were 7.2 pg/mL and 20.5 pg/mL, respectively. However, 7.8 pg/mL and 31.2 pg/mL were chosen as the nearest empirical values for LOD and LOQ, respectively.

### 2.2. Calculation of the African Lion Diagnostic Cut-Off Value for the QFT Mabtech Cat IGRA

**Figure 2:**
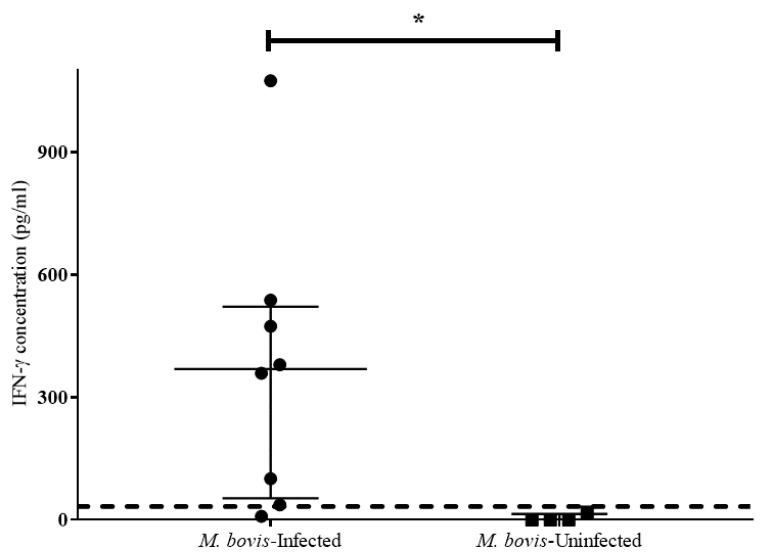
Comparison of antigen-specific interferon-gamma (IFN-γ) concentrations (pg/mL) in samples from *M. bovis*-infected (*n* = 8) and *M. bovis*-uninfected (*n* = 4) African lions using the QuantiFERON^®^-TB Gold Plus (QFT) Mabtech Cat IGRA. The Mann-Whitney U test showed a statistically significant difference between the results of these two cohorts (* *p* = 0.004). Medians and inter-quartile ranges are indicated by horizontal bars. The calculated assay cut-off value (33 pg/mL) is shown as a horizontal dotted line.

Using antigen-specific IFN-γ concentrations measured in QFT plasma samples from *M. bovis*-infected and uninfected lions, receiver operator characteristic (ROC) curve analysis was used to generate an African lion-specific cut-off value of 28 pg/mL (sensitivity = 87.5%; specificity = 100%), and an area under the curve (AUC) of 97% (Figure 2, Table 4). An alternative cut-off value of 33 pg/mL was calculated using the mean plus three standard deviations (mean + 3SD) of antigen-specific IFN-γ concentrations in samples from *M. bovis*-uninfected lions. This provided the same sensitivity (87.5%) and specificity (100%) as the cut-off value from the ROC curve analysis (Table 4).

### 2.3. Measurement of Antigen-Specific African Lion IFN-γ Concentrations in QFT Plasma

Mycobacterial antigen-specific IFN-γ concentrations measured using the QFT Mabtech Cat IGRA in *M. bovis*-infected and uninfected lions are shown in Figure 2. When antigen-specific IFN-γ concentrations were compared between *M. bovis*-infected (*n* = 8) (median 369.5 pg/mL, inter-quartile range (IQR) 469.0 pg/mL) and *M. bovis*-uninfected (*n* = 4) (median 0 pg/mL, IQR 14.2 pg/mL) individuals, there was a significant difference between the medians of these two groups (*p* = 0.004).

## 3. Discussion

The results of this study demonstrate the diagnostic potential of a commercially available IGRA for detecting *M. bovis* infection in African lions. Although IGRAs in lions have been previously described [15,17,18], this is the first study that reports results using TB antigen-stimulated whole-blood samples from culture-confirmed *M. bovis*-infected lions. To develop an assay using commercially available reagents, a domestic cat IFN-γ ELISA was screened to determine the cross-reactivity of antibodies with native lion IFN-γ in plasma from whole blood stimulated using the QFT platform. Due to the assay’s ability to measure significantly greater IFN-γ concentrations in mitogen-stimulated versus unstimulated African lion blood samples, provisional validation was performed. Importantly, the QFT Mabtech Cat IGRA had a preliminary calculated sensitivity and specificity of 87.5% and 100%, respectively, based on TB antigen-specific IFN-γ concentrations in culture-confirmed *M. bovis*-infected and uninfected lions, demonstrating its utility as a diagnostic assay. The preliminary diagnostic cut-off value was determined which resulted in high sensitivity and specificity, but with wide confidence intervals due to the small sample size.

Commercial anti-domestic cat IFN-γ antibodies (Mabtech) used in this study were able to recognise and bind to native African lion IFN-γ. This was likely due to the high homology (97–100%) between IFN-γ sequences isolated from cheetahs, lions and domestic cats [19]. A previous study has also shown cross-reactivity of the Mabtech antibodies with native cheetah IFN-γ [16]. Although a different supplier was used (R&D Systems, Inc., Minneapolis, MN, USA), a recent study showed that anti-cat IFN-γ antibodies could bind with rIFN-γ from five Felidae species, including lion, tiger (*Panthera tigris*), cheetah, cougar (*Puma concolor*), Iberian lynx (*Lynx pardinus*) and the Canadian lynx (*Lynx canadensis*) [17]. These findings suggest the presence of similar epitopes between different felid species’ cytokine molecules, specifically IFN-γ, which can be exploited to develop multi-species diagnostic tests.

The performance of the Mabtech Cat IFN-γ ELISA was evaluated using lion whole-blood samples stimulated using QFT tubes, to further assess the potential of the diagnostic test for detection of *M. bovis* infection in lions, and possibly other Felidae. This ELISA displayed good parallelism and linearity when comparing detection of domestic cat rIFN-γ with African lion IFN-γ, confirming the suitability of this assay for use with lion samples. The recovery of domestic cat rIFN-γ in the lion plasma matrix was acceptable for dilutions of 50% or more, due to minimal interference from other plasma components; therefore, a 1:4 sample dilution was selected [20]. In addition, the ability of the ELISA to provide consistent results was indicated by satisfactory intra- and inter-assay precision with CVs less than 10% [21]. Therefore, the Mabtech Cat IFN-γ ELISA^Basic^ kit was considered “fit-for-purpose” to measure African lion IFN-γ [22].

Using the QFT Mabtech Cat IGRA, there were significantly increased antigen-specific IFN-γ concentrations in plasma from the majority (6/8) of *M. bovis*-infected lions compared to the four uninfected animals, demonstrating the potential diagnostic value of this assay. Although limited samples from confirmed *M. bovis*-infected and uninfected lions were available, the calculation of a lion-specific diagnostic cut-off value using two methods was still possible. Since the lion diagnostic cut-off value calculated using ROC curve analysis (28 pg/mL) was below the LOQ of the ELISA, the value (33 pg/mL), calculated as the mean + 3SD [23], was selected as the preferred lion-specific cut-off value. This resulted in a relatively high estimated sensitivity and specificity for the lion QFT Mabtech Cat IGRA (87.5% and 100%, respectively) although the 95% CI was wide due to low sample size. The test performance of this IGRA was similar to IGRAs used in the domestic cat (68% sensitivity, 96% specificity), white rhinoceros (*Ceratotherium simum*) (78% sensitivity, 92% specificity), and African buffalo (89% sensitivity, 94% specificity) [24,25,26]. Therefore, future studies should confirm the diagnostic cut-off, sensitivity, and specificity of this IGRA using a larger sample size of lions.

Currently the only available tests for ante-mortem detection of *M. bovis* infection in lions are the TST [13] and the QFT *CXCL9* GEA [14]. Although the QFT *CXCL9* GEA is a useful alternative to the TST, since it requires only one immobilization for blood collection, the QFT Mabtech Cat IGRA is easier to use, can provide rapid results, and is more cost effective. In addition, the QFT Mabtech Cat IGRA makes use of commercially available antibodies and reagents which have several benefits. Firstly, it decreases costs and time associated with the development of species-specific reagents by research and veterinary diagnostic laboratories [27]. Secondly, the use of commercially available reagents allows for global access to reagents with better standardisation and comparison of results between research and veterinary diagnostic laboratories [28]. Importantly, the use of commercially available reagents which are cross-reactive in related species could lead to the development of multi-species diagnostic tests.

Although the QFT Mabtech Cat IGRA showed diagnostic potential for detecting *M. bovis* infection in lions, the limitation in this study was the small number of culture-confirmed *M. bovis*-infected and uninfected lions. Currently, the World Organisation for Animal Health (WOAH) requires samples from 30 known infected and 30 uninfected individuals for complete test validation in wildlife [22]. The performance of the QFT Mabtech Cat IGRA should be re-evaluated using a larger cohort of lions with confirmed infection status, especially since mycobacterial culture, the gold standard test that was used in this study to confirm *M. bovis* infection, is imperfect [29]. The use of QFT tubes, containing specific mycobacterial peptides (early secretory antigenic target 6 kDa and culture filtrate protein 10 kDa), for whole-blood stimulation may improve the specificity of the QFT Mabtech Cat IGRA compared with purified protein derivative (PPD)-based IGRAs (which make use of a crude antigen cocktail), although the logistical challenge of processing blood samples the same day of collection may not be feasible for the employment of either assay. However, pre-coated QFT vacutainer tubes allow for initial stimulation in the field if a portable incubator with a reliable power supply is available, which is more easily achieved compared to the preparation of the PPD-based stimulation system.

A previous study has shown that parallel testing can enhance the detection of *M. bovis*-infected animals [30], especially in the absence of a sensitive validated diagnostic test. Since many diagnostic tests have imperfect performance, parallel testing can improve diagnostic accuracy [31,32]. Although parallel tests can improve diagnostic sensitivity, some of the disadvantages include increased cost and a loss in specificity [30]. Therefore, future studies should compare the performance of the QFT Mabtech Cat IGRA with other available diagnostic tests for *M. bovis* detection in lions (TST; GEA), as well as consider the combined use of these tests. In addition, future studies should consider validating the QFT Mabtech Cat IGRA in other felid species if appropriate sample sizes are available. Confirmation of the cut-off value, sensitivity, and specificity of the assay using a larger cohort of *M. bovis*-infected, and *M. bovis*-uninfected African lions is recommended. Due to the challenges faced during development of species-specific bTB diagnostic assays in wildlife species, validating an assay for each species is critical to obtain reliable results. Therefore, this study demonstrates that validated pre-existing diagnostic tests for bTB can be adapted in related species to expand the diagnostic capacity for wildlife.

## 4. Materials and Methods

### 4.1. Animals and Sampling

Ante-mortem paired blood and post-mortem tissue samples (*n* = 12) were opportunistically collected from four free-ranging African lions in the Kruger National Park (KNP; Mpumalanga/Limpopo), and eight lions from private game reserves in Kwazulu-Natal (KZN), South Africa. An additional 25 lion blood samples, used for screening and preliminary validation, were collected in KNP. Sampling occurred during immobilization for management, disease surveillance, or veterinary procedures between 2017 and 2021. Tissue samples were only collected if animals were euthanized for humane reasons following veterinary assessment. Whole blood was collected into BD Vacutainer^®^ lithium heparin tubes (Becton, Dickinson and Company, Sparks, MD, USA), typically during night-time conditions (when lions were immobilized) and transported in a Styrofoam box to the laboratory at room temperature within eight hours of collection. Whole blood stimulation was performed by adding 1 mL aliquots of heparinised whole blood to a set of QuantiFERON^®^-TB Gold Plus (QFT) tubes (Qiagen, Hilden, Germany), as previously described [14,16]. The QFT tubes included a QFT nil tube (unstimulated negative control), QFT TB2 antigen tube containing specific mycobacterial peptides (ESAT-6 and CFP-10), and a QFT mitogen tube (positive control) containing phytohaemagglutinin (PHA). Pokeweed mitogen (PWM; 10 μg/mL final concentration in phosphate buffered saline (PBS); Sigma-Aldrich, St. Louis, MO, USA) was added to the QFT mitogen tube to ensure adequate stimulation. Tubes were then incubated for 24 h at 37 °C, and processed as previously described [14,16]. The *M. bovis* infection status of euthanized lions was confirmed using mycobacterial culture of post-mortem tissues [33], after which bacterial isolates were genetically speciated by genomic regions of difference PCR [34] to confirm *M. bovis* infection.

Immobilization of animals, blood collection, euthanasia, and tissue sampling were performed by South African Veterinary Council (SAVC)-registered wildlife veterinarians for procedures unrelated to this study.

### 4.2. Screening of the Mabtech Cat IFN-γ ELISA^Basic^ kit

The Mabtech Cat IFN-γ ELISA^Basic^ kit (catalogue no. 3122-1H-20; Mabtech AB, Nacka Strand, Sweden) was screened to determine if this commercially available kit could detect native African lion IFN-γ in QFT plasma. Plasma (QFT nil and mitogen) samples from four randomly selected African lions were pooled to create unstimulated (pooled QFT nil) and stimulated (pooled QFT mitogen) samples. Both pools were screened in duplicate wells at 1:2, 1:4, and 1:8 dilutions of lion plasma in incubation buffer/ELISA diluent (PBS, 0.05% Tween^®^20, ThermoFischer Scientific, Waltham, MA, USA; 0.1% bovine serum albumin). The ELISA kit protocol was performed following the manufacturer’s instructions. Briefly, a polystyrene microplate (ThermoFisher Scientific, Waltham, MA, USA) was coated with 100 µL per well of 2 µg/mL anti-cat IFN-γ monoclonal capture antibody (MT131) and incubated overnight at 4 °C. The plate was then washed twice with PBS (200 µL per well) before blocking each well with 200 µL of ELISA diluent and incubated for one hour at room temperature (22 °C). The plate was then washed five times using 300 µL per well of wash buffer (PBS, 0.05% Tween^®^20). A stock solution of 0.5 µg/mL domestic cat rIFN-γ was reconstituted and serially diluted (from 1:2–1:128) using ELISA diluent to produce a standard curve with concentrations that ranged from 7.81 pg/mL to 1000 pg/mL. An assay background control (negative control), consisting of ELISA diluent only, was also included. Both standards and samples were added to duplicate wells (100 µL per well), then incubated for two hours at room temperature. The plate was washed five times using 300 µL of wash buffer before adding 100 µL per well of 0.5 µg/mL biotinylated anti-cat IFN-γ monoclonal detection antibody (MT114). After one hour incubation at room temperature, the plate was washed five times before adding 100 µL per well of streptavidin-horseradish peroxidase (streptavidin-HRP, provided in the kit) diluted to 1:1000 in ELISA diluent. The plate was incubated for one hour at room temperature and washed five times prior to adding 100 µL per well of tetramethylbenzidine (TMB) (Becton, Dickinson and Company). Following incubation of the plate for 20 min at room temperature in the dark, 50 µL of stop solution (2M H_2_SO_4_) was added to each well. The OD of each well was measured at 450 nm and 630 nm using a Bio-Rad iMark^TM^ Microplate Absorbance Reader (Bio-Rad Laboratories, Inc., Hercules, CA, USA). For the calculation of results, the light absorbance of other materials was accounted for by subtracting OD_630nm_ from OD_450nm_ for each sample and standard curve wells, after which the mean OD (OD_450nm_–OD_630nm_) for each sample and standard was determined. The true OD results of a sample or standard were calculated by subtracting the mean OD of the negative control (background control) from the mean OD of samples or standards prior to calculating IFN-γ concentrations (pg/mL). The African lion sample IFN-γ concentrations were interpolated from ODs using a known concentration of recombinant domestic cat IFN-γ that was serially diluted according to the manufacturer’s protocol and assayed in duplicate to produce standard curves [35]. A four-parameter logistic (4PL) regression analysis was performed using GraphPad Prism 7 for Windows (version 7.04, GraphPad Software, Inc., San Diego, CA, USA; www.graphpad.com; accessed on 2 February 2022). The CV for sample dilutions was calculated and the optimal sample dilution for the assay was selected as the dilution at which the greatest differential response between QFT nil and QFT mitogen samples was observed. In addition, plasma from QFT nil, TB2 (antigen), and mitogen tubes from all sampled lions were screened with the Mabtech Cat IFN-γ ELISA^Basic^ kit, using the optimal sample dilution (1:4), for validation of the assay. Samples were tested in duplicate, and results calculated as above. Mycobacterial antigen-specific IFN-γ production for each lion was calculated as IFN-γ concentration (pg/mL) in QFT nil tube subtracted from IFN-γ concentration in QFT TB2 tube. Calculated antigen-specific IFN-γ concentrations were considered valid only if the mitogen response (IFN- γ concentration of QFT mitogen–QFT nil) was significantly greater than the unstimulated response (IFN- γ concentration of QFT nil).

### 4.3. Testing the Diagnostic Potential of the QFT Mabtech Cat IGRA

#### 4.3.1. Assay Linearity and Parallelism

Assay linearity and parallelism were evaluated using QFT mitogen samples from three African lions, previously tested with the Mabtech Cat IFN-γ ELISA^Basic^ kit, whose IFN-γ concentrations were less than 500 pg/mL (within the working range of the assay). Two-fold serial dilutions of each lion plasma sample were performed in ELISA diluent, starting at 1:2 up to 1:1024 (11-point series). The same 11-point dilution series was prepared using a stock solution of 0.5 µg/mL domestic cat rIFN-γ. All dilution series (lion plasma and cat rIFN-γ) samples were plated in duplicate, and relative IFN-γ concentrations measured and calculated as described above. Results of all dilution series were analysed using 4PL regression analysis (GraphPad Prism 7). For each dilution series, percentage recovery (%) at each dilution point was determined (measured IFN-γ concentrations/expected IFN-γ concentrations × 100%) and only dilution points with sample recoveries between 80–120% of the expected values were considered acceptable to determine the assay working range [20]. Assay linearity was assessed by calculating the correlation coefficient (R^2^) between concentrations of the domestic cat rIFN-γ and African lion IFN-γ dilution series. In addition, domestic cat rIFN-γ and African lion IFN-γ regression slope values were compared using an F-test [36] to determine parallelism of the assay.

#### 4.3.2. Spike and Recovery

Unstimulated African lion plasma samples (QFT nil), with low or no circulating IFN-γ, were selected to determine the recovery of domestic cat rIFN-γ in the lion plasma matrix. To achieve this, QFT nil plasma from 15 lions previously tested with the Mabtech Cat IFN-γ ELISA^Basic^ kit (ODs < 0.01) were pooled. Pooled plasma was then divided into 100% plasma, 50% plasma, 25% plasma, and 0% plasma matrix solutions using ELISA diluent. Reconstituted domestic cat rIFN-γ was spiked into the four plasma matrix dilutions (100%, 50%, 25%, and 0%) to achieve a final concentration of 80 pg/mL (expected rIFN-γ concentration). A matching unspiked sample for each plasma matrix dilution (100%, 50%, 25%, and 0%) was also prepared and all matrix dilutions (spiked and unspiked) were plated into triplicate wells using the Mabtech Cat IFN-γ ELISA^Basic^ kit as described above. The ODs of each well were measured, and IFN-γ concentrations (pg/mL) calculated with reference to the standard curve as mentioned previously. Percentage recovery (%) of spiked rIFN-γ from the plasma matrix dilutions was calculated as: [(rIFN-γ concentration in spiked samples - rIFN-γ in unspiked samples)/expected rIFN-γ concentration] × 100%.

#### 4.3.3. Assay Repeatability and Reproducibility

To evaluate assay repeatability and reproducibility, QFT mitogen samples previously tested with the Mabtech Cat IFN-γ ELISA^Basic^ kit, whose IFN-γ concentrations were known to be within the reported working range of the assay (<1000 pg/mL), were selected from three African lions. Nine aliquots of each QFT mitogen sample were prepared and stored at 4 °C during the testing period. Three aliquots per animal were assayed on the same plate over three consecutive days and mean IFN-γ concentrations (pg/mL) calculated. Intra-assay precision (within-run repeatability) was calculated as the mean CV between triplicates for each day. Inter-assay precision (between-run reproducibility) was calculated as the mean CV per sample over three days.

#### 4.3.4. Limit of Detection and Limit of Quantification

Limit of detection and limit of quantification of the assay were determined using 24 replicates of ELISA diluent. The mean OD and standard deviation (SD) values of replicates were calculated. The LOD was calculated as the mean OD + 3SD while LOQ was calculated as the mean OD + 10SD [37]. The OD values were then converted to IFN-γ concentrations (pg/mL) using the standard curve.

### 4.4. Statistical Analyses and Calculation of Diagnostic Cut-Off Value for QFT Mabtech Cat IGRA

Receiver operator characteristic (ROC) curve analysis was performed to determine an African lion-specific preliminary diagnostic cut-off value for the QFT Mabtech Cat IGRA. The ROC analysis was performed, using eight *M. bovis* culture-confirmed infected and four confirmed uninfected lions. The cut-off value with the highest Youden’s index was selected [38]. An alternative cut-off value was calculated, using the results from uninfected lions (*n* = 4), as the mean antigen-specific IFN-γ concentration plus three SDs [23]. A preliminary sensitivity was calculated as the number of true positive results (confirmed by mycobacterial culture) divided by the number of true positive + false negative results, while preliminary specificity was calculated as true negatives (confirmed by mycobacterial culture) divided by true negatives + false positives [39]. The median antigen-specific IFN-γ concentrations in QFT plasma from *M. bovis*-infected and *M. bovis*-uninfected African lions were compared using a Mann-Whitney U test. For all analyses, a *p*-value < 0.05 was considered statistically significant.

## Figures and Tables

**Table 1:** Interferon-gamma (IFN-γ) concentrations (pg/mL) of serially diluted pooled plasma from unstimulated (QFT nil) and stimulated (QFT mitogen) African lion whole-blood samples (*n* = 4) measured using the Mabtech Cat IFN-γ ELISA^Basic^ kit.

Recombinant IFN-γ Standard Range (pg/mL)	QFT Stimulation	Final Sample Concentration (pg/mL)			
		Sample Dilution			Mean ± SD
		1:2	1:4	1:8	
7.81–1000	nil	28	36	16	27 ± 10
	mit	1843	1943	1933	1906 ± 55
	mit–nil	1815	1907	1917	1880 ± 56

IFN-γ—interferon gamma; QFT—QuantiFERON^®^-TB Gold Plus; mit—QFT mitogen; SD—standard deviation.

**Table 2:** Recovery of 80 pg/mL domestic cat recombinant interferon gamma (rIFN-γ) spiked into pooled African lion plasma matrix (*n* = 15) and measured in triplicate using the Mabtech cat IFN-γ ELISA^Basic^ kit.

	Domestic Cat rIFN-γ Recovery (%)			
	100% Lion Plasma	50% Lion Plasma	25% Lion Plasma	0% Lion Plasma
Replicate 1	72	92	83	90
Replicate 2	63	85	88	99
Replicate 3	72	89	88	93
Mean	69.0	88.7	86.3	94.0
SD	5.2	3.5	2.9	4.6

rIFN-γ—recombinant interferon gamma; SD—standard deviation.

**Table 3:** Intra- and inter-assay precision of the Mabtech Cat IFN-γ ELISA^Basic^ kit calculated as the coefficient of variation (CV%) using plasma from QFT mitogen-stimulated whole blood from three African lions, measured in triplicate over a period of three consecutive days.

Animal ID	Intra-Assay Precision			Inter-Assay Precision		
	Mean IFN-γ Concentration (pg/mL)	SD	CV (%)	Mean IFN-γ Concentration (pg/mL)	SD	CV (%)
21/205	531.0	27.1	5.1	529.7	31.0	5.9
21/206	594.1	20.2	3.4	631.8	58.6	9.3
21/277	845.0	16.0	1.9	876.9	43.8	5.0

ID—identification number; IFN-γ—interferon gamma; QFT—QuantiFERON®-TB Gold Plus; SD—standard deviation; CV—coefficient of variation.

**Table 4:** Sensitivity and specificity of the QFT Mabtech Cat interferon-gamma (IFN-γ) release assay (IGRA) were calculated using samples from mycobacterial culture-confirmed *Mycobacterium bovis*-infected (*n* = 8) and *M. bovis*-uninfected (*n* = 4) African lions. A cut-off value for the assay was calculated using receiver operator characteristics (ROC) curve analysis, based on antigen-specific IFN-γ concentrations in samples from *M. bovis*-infected and uninfected lions. An alternative cut-off value was calculated using the mean + 3 standard deviations (SD) antigen-specific IFN-γ concentrations in samples from *M. bovis*-uninfected lions.

QFT Mabtech Cat IGRA					
Cut-Off Value (pg/mL)		Sensitivity (%)	95% CI	Specificity (%)	95% CI
ROC curve	28	87.5	47.4–99.7	100	39.8–100
Mean + 3 SD	33	87.5	47.4–99.7	100	39.8–100

CI—confidence interval; IFN-γ—interferon gamma; IGRA—interferon gamma release assay; *M. bovis*—*Mycobacterium bovis*; QFT—QuantiFERON^®^-TB Gold Plus; ROC—receiver operator characteristic; SD—standard deviation.

## Data Availability

The data presented in this study are available on request from the corresponding author.
